# A novel finding of hair growth like vellus hairs on glabrous skin of distal phalanx of thumb in Vogt–Koyanagi–Harada disease: A case report

**DOI:** 10.1097/MD.0000000000048170

**Published:** 2026-03-27

**Authors:** Muhammad Mateen Amir, Bilal Aslam, Umama Alam, Fathimathul Henna, Kamil Ahmad Kamil

**Affiliations:** a University of Lahore Teaching Hospital, Al-Khidmat Teaching Hospital, Lahore, Pakistan; b Opthalomology Department, University of Lahore Teaching Hospital, Lahore, Pakistan; c Department of Medicine, Khyber Medical College, Peshawar, Pakistan; d Department of Medicine, Dubai Medical College for Girls, Dubai, UAE; e Internal Medicine Department, Mirwais Regional Hospital, Kandahar, Afghanistan.

**Keywords:** alopecia, tinnitus, vellus hair, Vogt–Koynagi–Harada disease

## Abstract

**Rationale:**

This report describes a rare integumentary finding – vellus-like hair growth on the glabrous palmar skin – in a patient with VKHD. To our knowledge, such a manifestation has not been previously documented.

**Patient concerns:**

A 25-year-old woman presented with unusual hair growth on the radial and palmar surface of the distal thumb, emerging 2 years after the initial diagnosis of VKHD.

**Diagnoses:**

Previously diagnosed VKHD with recurrent episodes of auditory symptoms and rare occurrence of short, fine vellus-like hairs at a glabrous skin site.

**Interventions:**

She was managed with low-dose oral prednisolone (5–10 mg/d), which alleviated auditory symptoms. Dermatology consultation confirmed the unusual hair growth pattern.

**Outcomes:**

The hair growth recurred twice within a span of 2 years, lasting 4 to 6 weeks each time, and did not recur thereafter.

**Lessons:**

The presence of hair follicles or vellus-like hairs on glabrous skin is exceedingly rare. This observation expands the spectrum of integumentary manifestations associated with VKHD and warrants documentation for future reference.

## 1. Introduction

Alfred Vogt in 1906 followed by Koynagi in 1911 and then Harada in 1923 published cases with description of Vogt–Koynagai–Harada disease (VKHD). Harada described the posterior disease as acute posterior choroiditis.^[1]^ It is a multisystem autoimmune disorder that affects melanocyte-containing organs such as the eye, central nervous system, skin and auditory system.^[2]^ Age group is between 20 and 50 years, but it may occur in children and in old age.^[3,4]^

In the acute VKHD there are prodromal symptoms such as headache, tinnitus or vertigo followed by non- granulomatous inflammation involving the whole uveal tract causing sudden visual deterioration. Bilateral multiple serous retinal detachment with variable severity developed.^[5]^

VKHD is associated with various skin findings in convalescent stage such as vitiligo of eyebrows, eyelashes and skin. Alopecia of scalp hair may occur temporarily.^[6]^

Israelsen et al, a dermatologist by using ultrahigh resolution optical coherence tomography detected vellus hairs on glabrous area of palm which were comparable with vellus hairs on cheek.^[7]^ This study provides the basis to report the finding which we observed in our patient.

## 2. Case presentation

**Figure 1.**
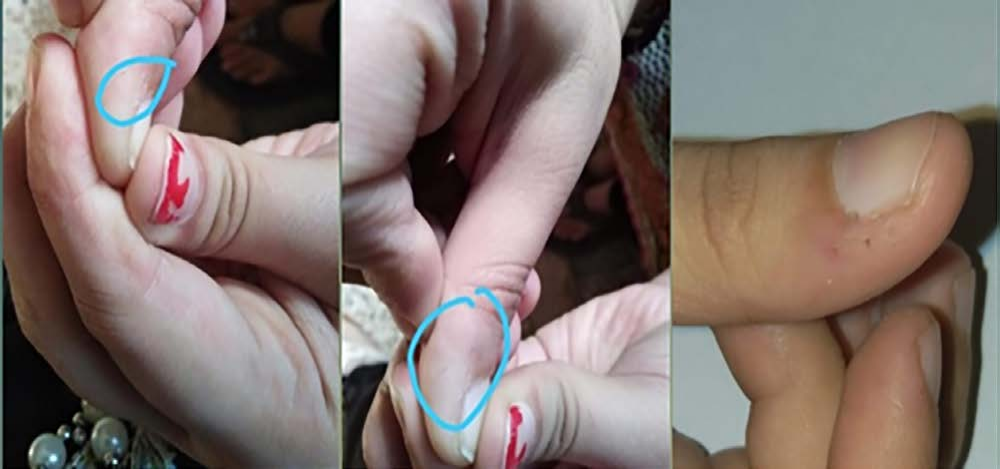
Photograph of fine, vellus-like hair on the radial and palmar surface of the distal phalanx of the right thumb.

This is a case that has been reported previously in 2017 by me and published in CPSP journal.^[5]^ A 25 year old female presented with the history of prodromal symptoms of headache and neck stiffness. On examination there was bilateral optic disc hyperemia and swelling mimicking the picture of optic neuritis. The patient later developed bilateral pan uveitis with serous retinal detachment. The case was treated successfully with posterior subtenon injection of triamcinolone. Later on in this patient an unusual finding developed which I am going to report for the first time. She came with the complaint of hair growth on radial and palmer surface of distal phalanx of thumb after 2 years in October 2019. Some of the hairs were near mucocutaneous junction. These hairs were fine, thin like vellus hairs (Fig. 1). Dermatologist was consulted for these integumentary changes. There was no history of hirsutism. This unique finding of vellus hairs came as a surprise as on extensive search of literature no such finding has been reported as yet in VKHD. She also developed tinnitus along with development of vellus hairs. The patient was on low dose steroids, prednisolone 5 mg/d. The auditory symptoms were relieved by increasing the dose to 10 mg/d. Whenever we stopped steroids the auditory symptoms recur and so the patient was kept on 5mg prednisolone per day for further 2 years. She didn’t develop glaucoma or cataract during treatment.

**Figure 2.**
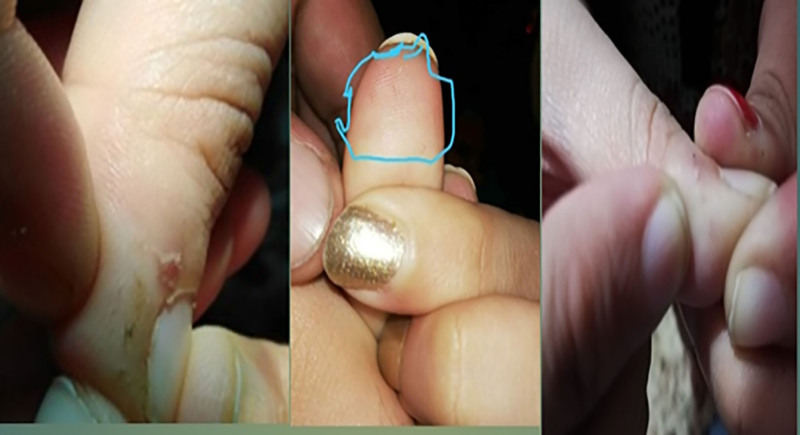
Recurrent appearance of similar vellus-like hair on the same site.

She again developed hair in June 2021 and came twice to show these hairs (Fig. 2). These were very fine short hair. The hair remained for 4 to 6 weeks. After that she never complained of hair growth again.

## 3. Discussion

Glabrous skin of palm and the papillary ridges is characterized by absence of hair follicles and sebaceous glands. Hairy skin complications are frequent in VKHD. Integumentary changes like alopecia, poliosis and vitiligo are late complications in this disease. The presence of vellus hairs on palm similar to cheeks have been demonstrated.^[7]^ Melanocytes are found in meninges and inner ear in addition to eye, skin and hairs. The pathogenesis of VKHD though not completely understood, involves auto antigens, the melanocyte differentiation proteins, like tyrosinase (TYR), tyrosinase-related peptide (TRP)-1, TRP-2.^[8,9]^ These proteins are the enzymes involved in the synthesis of melanin and are present specifically in melanocytes.^[10]^ Additional auto antigens, like KU-MEL-1 and lens epithelium-derived growth factor have a role through an IgG-mediated mechanism.^[9]^ The genetic predisposition involves certain genetic or epigenetic factors resulting in immune dysregulations.^[11]^ The growth of hairs on palmer surface of distal phalanx of the thumb may be the result of auto antigens along with immune dysregulations.

## 4. Conclusion

Dermatological complication such as alopecia, poliosis and vitiligo in VKHD usually occurs late in the disease after 3 to 12 months. The growth of hair on side of thumb is a novel finding not mentioned in the literature. The case report is much needed to be a reference in future, if found by any other clinician.

## Author contributions

**Conceptualization:** Muhammad Mateen Amir.

**Data curation:** Muhammad Mateen Amir, Fathimathul Henna.

**Formal analysis:** Bilal Aslam.

**Investigation:** Muhammad Mateen Amir.

**Methodology:** Muhammad Mateen Amir.

**Project administration:** Kamil Ahmad Kamil.

**Resources:** Fathimathul Henna.

**Supervision:** Muhammad Mateen Amir.

**Validation:** Muhammad Mateen Amir.

**Visualization:** Umama Alam.

**Writing – review & editing:** Bilal Aslam, Kamil Ahmad Kamil.

**Writing – original draft:** Muhammad Mateen Amir, Umama Alam.
